# Thromboelastometry in patients with advanced chronic liver disease stratified by severity of portal hypertension

**DOI:** 10.1007/s12072-020-10093-3

**Published:** 2020-09-30

**Authors:** Pierre Raeven, Joanna Baron-Stefaniak, Benedikt Simbrunner, Alexander Stadlmann, Philipp Schwabl, Bernhard Scheiner, Eva Schaden, Ernst Eigenbauer, Peter Quehenberger, Mattias Mandorfer, David Marek Baron, Thomas Reiberger

**Affiliations:** 1 Department of Anaesthesia, Intensive Care Medicine and Pain Medicine, Medical University of Vienna, Vienna, Austria; 2 Vienna Hepatic Hemodynamic Lab, Medical University of Vienna, Vienna, Austria; 3 Division of Gastroenterology and Hepatology, Department of Medicine III, Medical University of Vienna, Waehringer Guertel 18-20, 1090 Vienna, Austria; 4 IT4Science, Medical University of Vienna, Vienna, Austria; 5 Department of Laboratory Medicine, Medical University of Vienna, Vienna, Austria

**Keywords:** Cirrhosis, Hepatic venous pressure gradient, Varices, Ascites, Viscoelastic test, Coagulation, Bacterial translocation, Inflammation, Clinical, Hepatic

## Abstract

**Background:**

Rotational thromboelastometry (ROTEM) has been studied in patients with advanced chronic liver disease (ACLD) without considering the impact of portal hypertension. We evaluated the influence of the hepatic venous pressure gradient (HVPG) on ROTEM results in patients with ACLD.

**Methods:**

Cross-sectional study; ACLD patients undergoing HVPG measurement within the prospective Vienna Cirrhosis Study (NCT03267615) underwent concomitant ROTEM testing.

**Results:**

Among 159 patients (68% male; Child–Pugh-A: 53%, Child–Pugh-B: 34%, Child–Pugh-C: 13%), 21 patients (13%) had a HVPG between 6 and 10 mmHg, 84 patients (53%) between 10 and 19 mmHg, and 54 patients (34%) ≥ 20 mmHg. Child–Pugh-C patients (vs. Child–Pugh-A and vs. Child–Pugh-B patients, respectively) showed longer clot formation time (CFT: median 187 s vs. 122 s vs. 122 s, *p* = 0.007) and lower maximum clot firmness (MCF: median: 45 mm vs. 56 mm vs. 56 mm, *p* = 0.002) in extrinsic thromboelastometry (EXTEM), while platelet counts were similar across Child–Pugh stages. In the overall cohort, ROTEM parameters did not differ by severity of portal hypertension. However, among compensated Child–Pugh-A patients, MCF decreased with increasing portal pressure, i.e. in higher HVPG strata (HVPG 9–10 mmHg: median MCF: 59 mm vs. HVPG 10–19 mmHg: 56 mm vs HVPG ≥ 20 mmHg: 54 mm, *p* = 0.023). Furthermore, patients with short CFT and high MCF in EXTEM had higher levels of lipopolysaccharide-binding protein, C-reactive protein, and procalcitonin, as well as higher leukocyte counts (all *p* < 0.05).

**Conclusions:**

Portal hypertension seems to impact ROTEM results only in compensated Child–Pugh-A patients. Bacterial translocation and systemic inflammation may trigger a procoagulant state in patients with ACLD.

**Electronic supplementary material:**

The online version of this article (10.1007/s12072-020-10093-3) contains supplementary material, which is available to authorized users.

## Introduction

Assessment of hemostasis is challenging in patients with advanced chronic liver disease (ACLD) [1]. Prothrombin time-derived international normalized ratio (PT-INR) and activated partial thromboplastin time (aPTT) are often used to evaluate hemostasis and coagulation. Although PT-INR reflects liver synthetic function and is integrated into the Child–Pugh score and Model for end-stage liver disease (MELD), it does not indicate bleeding risk in ACLD patients [2]. This is explained by a concomitant decrease of anti-coagulant factors not captured by PT-INR (e.g. antithrombin, protein C, thrombomodulin) and increased levels of von Willebrand Factor and factor VIII [3]. Similarly, aPTT may detect coagulation factor deficiencies or guide heparin therapy but is not associated with disturbed hemostasis in ACLD patients. Of note, PT-INR and aPTT are based on clot formation as an endpoint, which occurs when approximately 5% of thrombin is generated [4].

Rotational thromboelastometry (ROTEM) is a global viscoelastic coagulation test, validated to guide the treatment of severe bleeding in trauma or major surgical procedures. As a point-of-care test using whole blood, ROTEM represents a more comprehensive test to assess the hemostatic status. Several studies have investigated the use of viscoelastic tests in patients with ACLD from different perspectives [5–10]. Of note, these studies did not assess the influence of hepatic venous pressure gradient (HVPG) or report on the prevalence of clinically significant portal hypertension (CSPH).

CSPH is defined as HVPG ≥ 10 mmHg [11], and is associated with the development of varices and the progression of compensated ACLD (cACLD) to decompensated ACLD [12, 13, 14]. Moreover, HVPG ≥ 20 mmHg is associated with high risk of bleeding-related mortality [15, 16].

In ACLD, CSPH and liver dysfunction facilitate bacterial translocation from the gut to the splanchnic and systemic circulation [17, 18]. This process can trigger systemic inflammation, reflected by increased circulating levels of lipopolysaccharide-binding protein (LBP), interleukin-6, C-reactive protein (CRP), and procalcitonin, as well as elevated leukocyte counts [17]. Importantly, activation of hemostasis may be a physiological response to bacterial translocation, possibly limiting the dissemination of pathogens [19].

In this study, we examined (i) whether the severity of portal hypertension and hepatic dysfunction affect hemostasis as assessed by ROTEM and (ii) if ROTEM results are associated with parameters related to bacterial translocation.

## Patients and methods

### Patient selection

Data were derived from the prospective Vienna Cirrhosis Study (VICIS). Inclusion criteria were age ≥ 18 years, diagnosis of ACLD (liver stiffness > 15 kPa, HVPG > 5 mmHg, or histologic F3/F4 fibrosis) and written informed consent. Data sets collected from 336 patients between June 2017 and May 2019 were screened. Exclusion criteria were missing/inconclusive HVPG measurements, missing ROTEM results, presence of acute decompensation, severe alcoholic hepatitis, acute-on-chronic liver failure, hepatocellular carcinoma, liver metastasis, congestive heart failure, previous liver transplantation or transjugular intrahepatic portosystemic shunt. Finally, 159 patients were included in the current study.

Patients under non-selective beta-blockers (NSBB) treatment (*n* = 30) were included in the analysis, as we expected a limited effect of NSBB on ROTEM. NSBB were paused in 26 patients 5 days prior to HVPG measurement. The interruption of NSBB treatment for baseline HVPG measurement is a routine clinical practice at our institution, we are not aware of any bleeding events that occurred in this context. The safety of this approach is also supported by previous studies [20]. Anti-coagulant/anti-platelet medication was paused for HVPG measurements.

### Measurement of the hepatic venous pressure gradient (HVPG)

Hepatic hemodynamic characterization was performed at the Vienna Hepatic Hemodynamic Lab of the Medical University of Vienna in accordance with a standardized operating procedure [21].

### Clinical and laboratory parameters

Demographic characteristics and blood samples were obtained on the day of the HVPG assessment. Child–Pugh score and MELD were calculated based on patients’ medical history and laboratory parameters. ROTEM was performed at the Department of Anesthesia, all other laboratory analyses at the Department of Laboratory Medicine (both Medical University of Vienna). Complete blood count and standard biochemical, coagulation, and immunological parameters were analyzed by routine methods.

### Rotational thromboelastometry (ROTEM)

Viscoelastic testing was performed with blood drawn into sodium citrate tubes using the ROTEM delta device (TEM International, Munich, Germany). In the intrinsic thromboelastometry (INTEM), the intrinsic coagulation pathway was activated by ellagic acid, whereas in the extrinsic thromboelastometry (EXTEM), recombinant tissue factor was added to whole blood. In the fibrinogen thromboelastometry (FIBTEM), fibrin polymerization was assessed by adding the platelet inhibitor cytochalasin D to the EXTEM test. We assessed clotting time (CT), defined as the time in seconds until clot initiation; clot formation time (CFT), which indicates the time in seconds from initial clotting until an amplitude of 20 mm is reached; and maximum clot firmness (MCF), which shows the greatest amplitude. For FIBTEM, only MCF was assessed.

### Stratification of patients

Patients were stratified by the severity of liver dysfunction and of portal hypertension into prognostic stages that reflect the risk of mortality. With compensated (cACLD) patients, we discriminated three substages [22]: stage 0 (subclinical portal hypertension [HVPG 6–9] mmHg, no varices), stage 1 (CSPH without varices), and stage 2 (presence of varices). In addition, to explore the correlation of bacterial translocation with ROTEM results at both ends of the distribution, we stratified our cohort into quintiles according to ROTEM values: 0–20% percentile (Q1), 20–80% percentile (Q2–4), 80–100% percentile (Q5).

### Statistics

GraphPad Prism 8 (GraphPad Software, La Jolla, CA, USA) and IBM SPSS Statistics 25 (IBM, Armonk, NY, USA) were used for statistical analyses. Patient characteristics were analyzed using descriptive statistics. Continuous data were expressed as median (interquartile range), while categorical data were expressed as number (proportion) of patients with the specific characteristic. As appropriate, analysis of variance or Kruskal–Wallis test (with Tukey’s or Dunn’s correction for multiple comparisons), Student’s *t* test and Mann–Whitney *U* test were applied for group comparisons of continuous variables, while categorical variables were compared using Chi squared test. Correlations were expressed as Spearman’s rho. A *p*-value < 0.05 was defined as statistically significant.

## Results

### Patient characteristics (Table 1)

**Fig. 1:**
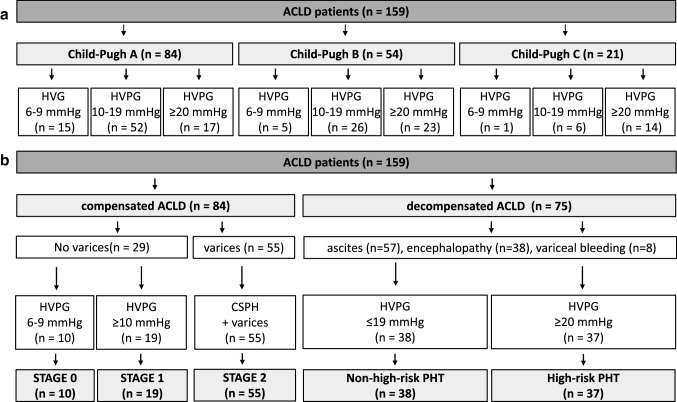
Patient flow chart. Patients were stratified based on Child–Pugh stage. **a** Within Child–Pugh subcohorts, patients were further grouped based on predefined HVPG cut-offs. **b** Patients with Child–Pugh-A were subdivided into stages 0, 1, and 2. Patients with Child–Pugh-B and -C were stratified based on the presence or absence of high-risk portal hypertension, as defined by a HVPG ≥ 20 mmHg. Some patients had more than one decompensating event. ACLD, advanced chronic liver disease; HVPG: hepatic-venous pressure gradient; PHT, portal hypertension

Patients were clustered based on the Child–Pugh stage and HVPG strata (Fig. 1a). Compensated patients were further grouped according to their prognostic stage (Fig. 1b). In addition, decompensated (i.e. Child–Pugh-B or -C) patients were characterized based on the presence of high-risk portal hypertension: while 51% of patients had HVPG values < 20 mmHg, the remaining 49% had HVPG values ≥ 20 mmHg (Fig. 1b).

**Table 1:** Patient characteristics^a^

Characteristics	*n* (%)
Patients	159 (100%)
Age (median years)	58
Sex (male)	108 (68%)
Etiology ALD Viral hepatitis ALD + viral hepatitis NASH Cholestatic Other Total	63 (40%) 38 (24%) 13 (8%) 14 (9%) 5 (3%) 26 (16%) 159 (100%)
HVPG 6–9 mmHg 10–19 mmHg ≥ 20 mmHg	21 (13%) 84 (53%) 54 (34%)
Child–Pugh stage A B C	84 (53%) 54 (34%) 21 (13%)
MELD Natrium score	13 [10–17]
MELD UNOS score	10 [9–15]
PT-INR	1.4 [1.2–1.6]
Albumin (g/l)	36 [32–40]
Bilirubin (mg/dl)	1.1 [0.7–2.0]
Creatinine (mg/dl)	0.7 [0.6–1.0]

^a^Continuous data are expressed as medians with interquartile range in square brackets. Categorical data are expressed as the number of patients with the specific characteristic (with the percentage in brackets). ALD, alcoholic liver disease; NASH, non-alcoholic steatohepatitis; HVPG, hepatic venous pressure gradient; MELD, Model for end-stage liver disease; UNOS, United Network for Organ Sharing; PT-INR, prothrombin time-derived international normalized ratio

### ROTEM results according to Child–Pugh stages (Table 2)

Initially, selected ROTEM parameters, plasma fibrinogen levels, and platelet counts were evaluated according to the Child–Pugh stage (Fig. 1a). In EXTEM, patients with Child–Pugh-C had prolonged CFT (+14% vs. Child–Pugh-A, *p* = 0.026; + 15% vs. Child–Pugh-B, *p* = 0.005) and reduced MCF (– 15% vs. Child–Pugh-A, *p* = 0.004; − 16% vs. Child–Pugh-B, *p* = 0.002). Child–Pugh-C patients had prolonged CT (+19% vs. Child–Pugh-A, *p* = 0.011) and CFT (+49% vs. Child–Pugh-B, *p* = 0.009) in INTEM and decreased MCF in INTEM (– 15% vs. Child–Pugh-A, *p* = 0.001; − 17% vs. Child–Pugh-B, *p* < 0.001) and FIBTEM (− 33% vs. Child–Pugh-A; − 40% vs. Child–Pugh-B, both *p* < 0.001). Simultaneously, fibrinogen levels decreased by 35% and 32% (both *p* < 0.001) in Child–Pugh-C vs. Child–Pugh-A and Child–Pugh-B patients, respectively. Taken together, severe ACLD (Child–Pugh stage C, as compared to stages A or B) was associated with slower clot initiation and formation, attenuated clot firmness, and lower fibrinogen levels, but not with reduced platelet count.

**Table 2:** Results of selected ROTEM tests, platelet counts and fibrinogen levels in patients stratified by Child–Pugh status^a^

	Child–Pugh A	Child–Pugh B	Child–Pugh C	*p*-value
EXTEM CT (s)	74 [66–89]	75 [66–82]	74 [70–93]	0.463
EXTEM CFT (s)	122 [99–200]*	122 [82–188]**	187 [133–255]	0.007
EXTEM MCF (mm)	56 [49–59]**	56 [49–62]**	45 [41–52]	0.002
INTEM CT (s)	192 [170–217]*	201 [169–224]	227 [191–253]	0.014
INTEM CFT (s)	105 [82–173]	105 [69–152]*	163 [107–214]	0.012
INTEM MCF (mm)	55 [49–59]**	55 [46–61]***	44 [40–51]	< 0.001
FIBTEM MCF (mm)	14 [11–18]***	15 [12–20]***	10 [7–13]	< 0.001
Fibrinogen (mg/dl)	288 [238–343]***	274 [219–365]***	186 [142–235]	< 0.001
Platelets (G/µl)	99 [65–134]	106 [71–156]	91 [56–102]	0.122

^a^Data are expressed as medians with interquartile range in square brackets. *n* = 84 (Child–Pugh A), *n* = 54 (Child–Pugh B, except *n* = 53 for platelet count), *n* = 21 (Child–Pugh C). Data were analyzed using analysis of variance (ANOVA) with Tukey’s test for post hoc comparisons (INTEM MCF and fibrinogen) or Kruskal–Wallis test with Dunn’s test for post hoc comparisons (remaining parameters). ROTEM, rotational thromboelastometry; EXTEM, extrinsic thromboelastometry; INTEM, intrinsic thromboelastometry; CT, clotting time; CFT, clot formation time; MCF, maximal clot firmness; FIBTEM, fibrinogen thromboelastometry. *p < 0.05 vs. Child–Pugh C, **p < 0.01 vs. Child–Pugh C, ***p < 0.001 vs. Child–Pugh C

### ROTEM results according to HVPG strata in compensated and decompensated ACLD

We then analyzed selected ROTEM parameters, plasma fibrinogen levels, and platelet counts according to HVPG strata (Fig. 1a). As these parameters were similar across different HVPG strata in the overall cohort (Table 3), we specifically examined whether HVPG impact on ROTEM results in cACLD patients (Table 4). Within cACLD patients, MCF in EXTEM and INTEM and platelet counts progressively decreased with the severity of portal hypertension (Table 4).

**Table 3:** Results of selected ROTEM tests, platelet counts and fibrinogen levels in patients stratified by severity of portal hypertension^a^

	HVPG			
	6–9 mmHg	10–19 mmHg	≥ 20 mmHg	*p*-value
EXTEM CT (s)	77 [69–87]	74 [66–85]	75 [65–88]	0.656
EXTEM CFT (s)	126 [88–174]	128 [100–208]	132 [90–219]	0.804
EXTEM MCF (mm)	57 [50–65]	55 [46–59]	53 [44–60]	0.347
INTEM CT (s)	200 [167–233]	198 [169–222]	203 [180–227]	0.561
INTEM CFT (s)	103 [75–144]	110 [82–175]	114 [75–201]	0.653
INTEM MCF (mm)	55 [52–64]	55 [45–59]	52 [44–59]	0.271
FIBTEM MCF (mm)	13 [11–21]	14 [10–16]	14 [10–18]	0.921
Fibrinogen (mg/dl)	313 [201–373]	261 [216–324]	273 [209–341]	0.323
Platelets (G/µl)	141 [74–164]	99 [67–132]	96 [55–126]	0.173

^a^Data are expressed as medians with interquartile range in square brackets. *n* = 21 (HVPG 6–9 mmHg), *n* = 84 (HVPG 10–19 mmHg, except *n* = 83 for platelet count), *n* = 54 (HVPG ≥ 20 mmHg). Data were analyzed using analysis of variance (ANOVA) with Tukey’s test for post hoc comparisons (EXTEM MCF, INTEM MCF and fibrinogen) or Kruskal–Wallis test with Dunn’s test for post hoc comparisons (remaining parameters). ROTEM, rotational thromboelastometry; HVPG, hepatic venous pressure gradient; EXTEM, extrinsic thromboelastometry; INTEM, intrinsic thromboelastometry; CT, clotting time; CFT, clot formation time; MCF, maximal clot firmness; FIBTEM, fibrinogen thromboelastometry

**Table 4:** Results of selected ROTEM tests, platelet counts and fibrinogen levels in compensated (Child–Pugh A) patients stratified by severity of portal hypertension^a^

	HVPG			
	6–9 mmHg	10–19 mmHg	≥ 20 mmHg	*p* value
EXTEM CT (s)	71 [66–90]	74 [66–87]	76 [65–93]	0.895
EXTEM CFT (s)	109 [80–152]	121 [102–216]	127 [99–232]	0.212
EXTEM MCF (mm)	59 [54–68]	56 [48–59]*	54 [45–58]*	0.023
INTEM CT (s)	181 [152–215]	192 [168–214]	198 [177–238]	0.280
INTEM CFT (s)	101 [62–118]	105 [83–175]	118 [91–220]	0.109
INTEM MCF (mm)	57 [53–67]	55 [48–59] *	52 [44–55] *	0.009
FIBTEM MCF (mm)	17 [12–23]	13 [11–16]	14 [12–18]	0.066
Fibrinogen (mg/dl)	338 [262–388]	281 [229–330]	289 [235–335]	0.057
Platelets (G/µl)	143 [88–169]	99 [65–132] *	84 [40–117] *	0.002

^a^Data are expressed as medians with interquartile range in square brackets. *n* = 15 (HVPG 6–9 mmHg), *n* = 52 (HVPG 10–19 mmHg), *n* = 17 (HVPG ≥ 20 mmHg). Data were analyzed using analysis of variance (ANOVA) with Tukey’s test for post hoc comparisons (EXTEM MCF, INTEM CT, INTEM MCF, FIBTEM MCF) or Kruskal–Wallis test with Dunn’s test for post hoc comparisons (remaining parameters). ROTEM, rotational thromboelastometry; HVPG, hepatic venous pressure gradient; EXTEM, extrinsic thromboelastometry; INTEM, intrinsic thromboelastometry; CT, clotting time; CFT, clot formation time; MCF, maximal clot firmness; FIBTEM, fibrinogen thromboelastometry. *p < 0.05 vs. HVPG 6–9 mmHg

In contrast, within decompensated (Child–Pugh-B/C patients, Table-S2) ACLD patients, ROTEM, fibrinogen levels, and platelet counts were similar across HVPG strata. Similarly, there was no difference in ROTEM results, fibrinogen levels or platelet counts in patients on NSBB (Table-S3) or between subcohorts stratified by severity of portal hypertension (Table-S4).

### ROTEM results in patients with various prognostic stages of portal hypertension

We then analyzed ROTEM results in compensated ACLD patients across substages based on the presence of varices and severity of portal hypertension (Fig. 1b). Although there was a trend towards prolonged CFT in EXTEM and INTEM and impaired MCF in EXTEM, INTEM, and FIBTEM in more advanced compensated ACLD, only INTEM CT was significantly prolonged by 17% (*p* = 0.045) in stage 2 compared to stage 0 patients (Table-S5). This increase in INTEM CT was associated with decreased platelet counts in stage 2 (− 40%, *p* < 0.001) and stage 1 (− 17%, *p* = 0.033) compared to stage 0 cirrhotic patients (Table-S5). Of note, aPTT (stage 0: 35 s; stage 1: 38 s; stage 2: 39 s; *p* = 0.099) was not different between early compensated stages of ACLD.

### ROTEM results in decompensated ACLD with or without high-risk portal hypertension

ROTEM values were compared among dACLD patients stratified by the presence or absence of high-risk portal hypertension (i.e. HVPG ≥ 20 mmHg, Fig. 1b). Within the dACLD population, all ROTEM analyses, as well as fibrinogen levels and platelet counts, were not significantly different regardless of the presence of high-risk portal hypertension (Table-S6). This may indicate that within dACLD patients, the degree of liver dysfunction affects ROTEM results to a greater extent than the severity of portal hypertension.

### Correlations of ROTEM results with clinical scores and laboratory parameters

Table-S7 shows a crude correlation matrix of analyzed parameters and ROTEM parameters. Importantly, ROTEM parameters did not correlate with Child–Pugh score and MELD, nor with HVPG or liver stiffness. Fibrinogen levels and platelet counts showed the strongest correlation with MCF and CFT, but not with CT. Concentrations of LBP and leukocyte counts showed a weak to moderate correlation with CFT and MCF in EXTEM, INTEM, and FIBTEM. Levels of CRP were correlated with CFT in EXTEM and INTEM as well as with MCF in FIBTEM.

### Analysis of the potential impact of bacterial translocation and systemic inflammation on ROTEM results

**Fig. 2:**
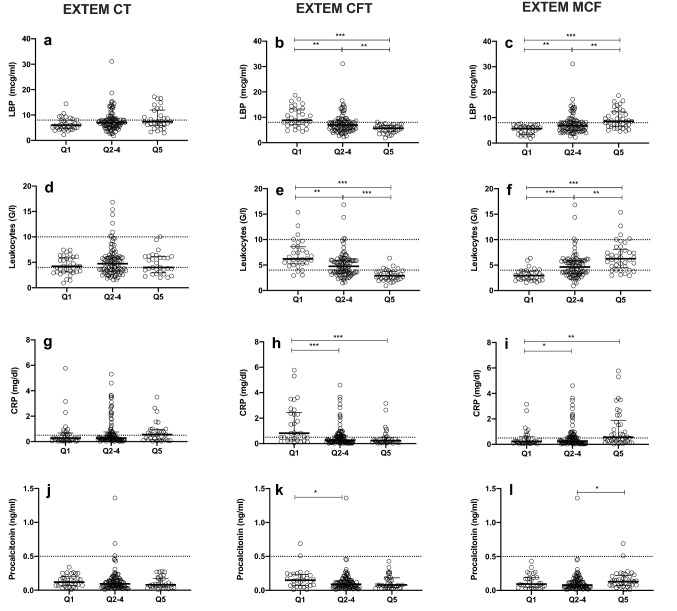
Impact of bacterial translocation and systemic inflammation on extrinsic thromboelastometry results. Lipopolysaccharide-binding protein in **a** EXTEM CT, **b** EXTEM CFT and **c** EXTEM MCF strata. Leukocytes in **d** EXTEM CT, **e** EXTEM CFT and **f** EXTEM MCF strata. C-reactive protein levels in **g** EXTEM CT, **h** EXTEM CFT and **i** EXTEM MCF strata. Procalcitonin levels in **j** EXTEM CT, **k** EXTEM CFT and (L) EXTEM MCF strata. *p < 0.05; **p < 0.01; ***p < 0.001 (analysis of variance/Tukey’s or Kruskal–Wallis/Dunn’s). Dotted lines indicate (lower and) upper reference value according to manufacturer/laboratory. EXTEM, extrinsic thromboelastometry; CT, clotting time; CFT, clot formation time; MCF, maximum clot firmness. LPB, lipopolysaccharide-binding protein; CRP, C-reactive protein; Q1, 0–20 percentile; Q2–4, 21–80 percentile; Q5, 81–100 percentile

Based on the results observed in the correlation matrix, we evaluated whether patients with ROTEM values in the lowest (Q1) and highest (Q5) quintiles differ in surrogate parameters of bacterial translocation and systemic inflammation (Fig. 2a–l and Fig.S1). Higher levels of LBP (Fig. 2a–c) and higher leukocyte counts (Fig. 2d–f) were associated with shorter CFT and stronger MCF in EXTEM (*p* < 0.05). The impact of systemic inflammation on ROTEM results was underlined by a similar pattern for CRP and procalcitonin (Fig. 2g–l) as for leukocyte count.

We further analyzed these parameters of bacterial translocation and systemic inflammation in the whole cohort and in Child–Pugh B/C patients with or without high-risk portal hypertension (Tables-S8, S9). In the overall cohort, CRP was significantly higher in the patients with high-risk (HVPG ≥ 20 mmHg) versus non-high-risk (HVPG 6-19 mmHg) portal hypertension, but all other parameters were not significantly different.

Of note, 20 patients were treated with rifaximin for encephalopathy, two received antibiotic treatment for secondary prophylaxis after spontaneous bacterial peritonitis (ciprofloxacin and norfloxacin), one received amoxicillin/clavulanic acid for urinary tract infection and one received trimethoprim/sulfamethoxazole for pneumocystis jirovecii pneumonia prophylaxis.

Taken together, these data indicate that a systemic proinflammatory state and bacterial translocation—often present in patients with cirrhosis—potentially affect clot formation and clot firmness as assessed by ROTEM.

## Discussion

To our knowledge, this is the first study investigating the influence of HVPG on ROTEM parameters in a thoroughly characterized ACLD cohort. First, observed that severe liver dysfunction and low fibrinogen levels are associated with prolonged clot formation and reduced clot firmness. Importantly, by the use of HVPG measurements, we could demonstrate that portal hypertension impairs clot firmness only in compensated patients. In addition, our ROTEM results suggest that pathological bacterial translocation and systemic inflammation are associated with shorter CFT and enhanced MCF and may predispose to a procoagulant state in patients with ACLD.

In our cohort, 87% of patients had CSPH (HVPG ≥ 10 mmHg), and a considerable number of patients had decompensated disease (47% with Child–Pugh B/C), indicating more advanced disease as compared to other ACLD patient cohorts [13]. Moreover, a thorough characterisation of HVPG distinguishes our data from previous studies assessing ROTEM [5, 7–10] or thromboelastography (TEG) [6], which did not report HVPG and included mostly compensated patients.

Interestingly, ROTEM results were not associated with the degree of portal hypertension in the entire cohort. In contrast, increased portal pressure was associated with decreased MCF in compensated Child–Pugh-A patients. This finding may be causally related to the coinciding decrease in platelet counts due to hypersplenism secondary to portal hypertension. However, low platelet counts are not associated with increased bleeding risk in cACLD patients, even in those on anti-coagulant medication [23]. Interestingly, a comparable degree of thrombocytopenia was observed across three Child–Pugh stages. This might be explained by a referral bias since among Child–Pugh A patients, those with thrombocytopenia are considerably more likely to be referred to HVPG measurement. Of note, about half of the Child–Pugh-A patients with HVPG ≥ 20 mmHg were previously decompensated. Although HVPG values ≥ 16–20 mmHg are predictive of the development of hepatic decompensation in compensated patients [21, 24], HVPG values may exceed ≥ 20 mmHg in compensated patients. For instance, Jindal et al. reported that 19% of compensated patients had HVPG values ≥ 20 mmHg [24]. To further elucidate the potential impact of portal hypertension on coagulation in compensated patients, we also integrated the presence of varices into our analysis. Therefore, we stratified cACLD patients into substage 0 without CSPH, substage 1 with CSPH but without varices, and substage 2 with CSPH and with varices [22]. Again, a trend towards impaired clot formation was observed from substage 0 to substage 2 of portal hypertension, and lower platelets in substages 1/2 compared to substage 0. Interestingly, cACLD patients in substage 2 showed a significantly prolonged clotting time in INTEM. There are various potential reasons for this observation: First, this could be an effect of endogenous heparinoids. Second, a prolonged INTEM CT in substage 2 might have been influenced by decreased clotting factors. Previous studies indicate that blood tests of synthetic function are linked to the severity of portal hypertension and models including INR have been shown to indicate the presence of CSPH in cACLD patients [25]. However, all of these “easily-available” markers are in many ways inaccurate and the diagnostic and discriminatory value for CSPH (vs. no CSPH) is only moderate. Other—more robust non-invasive markers—are currently being investigated but are not yet applied clinically. While the severity of the liver disease is usually reflected by both HVPG (i.e., extent of portal hypertension) and hepatic impairment, these parameters do not always match in their severity. Furthermore, there is no conclusive evidence that severity of portal hypertension per se impacts on hepatic synthetic function. However, portal hypertension drives bacterial translocation into the systemic circulation, which likely impacts hepatic function by triggering a pro-inflammatory response [18]. Accordingly, the reduced hepatic synthetic function could have contributed to the tendency towards impaired INTEM in stage 1/2 patients. Third, this observation might be the result of a type 1 error.

We then assessed ROTEM results within patients with the most advanced liver disease. Along with Child–Pugh-C and active bleeding at endoscopy, an HVPG of ≥ 20 mmHg is associated with failure to control variceal bleeding and bleeding-related mortality, thus defining high-risk portal hypertension [15]. In contrast to observations in compensated patients, ROTEM results did not differ among decompensated patients with various degrees of portal hypertension (i.e. between HVPG < 20 mmHg vs. ≥ 20 mmHg). Interestingly, platelet count and fibrinogen levels were not affected by HVPG status. This observation might be explained by a more pronounced impact of biochemical (i.e. impaired synthesis of determinants of hemostasis) rather than mechanical (i.e. portal hypertension) factors on ROTEM results. Decreased thrombopoietin production in dACLD patients may have ameliorated the impact of portal hypertension on platelet counts. However, in our study, their thrombopoietin levels were not significantly different between HVPG subgroups (data not shown).

In clinical practice, ROTEM may not be useful as a predictive tool for (spontaneous) or iatrogenic bleeding, but more as an asset to guide the use of blood products in case of ongoing bleeding. Nevertheless, a hypocoagulable state detected by ROTEM/TEG has been shown in patients who experienced early variceal rebleeding [26] and bleeding during invasive procedures [27].

Since there were few patients across all Child–Pugh stages and all HVPG strata who showed unexpectedly low or high results on ROTEM, we also assessed potential mechanistic factors for this phenomenon. Interestingly, we found that parameters of bacterial translocation (LBP) and systemic inflammation (i.e. leukocyte counts, CRP and PCT) inversely correlated with CFT and positively correlated with MCF. This potential impact of bacterial translocation on ROTEM is especially relevant, since bacterial translocation in ACLD patients is driven by portal hypertension [17] and by liver dysfunction. However, parameters of bacterial translocation and systemic inflammation—except for CRP levels—were similar in patients with low-risk versus high-risk portal hypertension.

We have recently observed a positive correlation between von Willebrand Factor antigen levels as an indicator of endothelial dysfunction and markers of systemic inflammation, independent of HVPG [28]. Elevated thrombin generation capacity and an unbalanced ratio between von Willebrand factor and its cleaving protein (ADAMTS13) have recently been described in cirrhosis [29]. Moreover, increases in CRP are paralleled by increases in factor VIII in patients with ACLD/portal hypertension without active bacterial infection [3]. Overall, this would suggest that bacterial translocation and a proinflammatory state result in a shorter clot formation time and stronger clot firmness, i.e. (inappropriate) promotion of clot formation and stability [30]. Even if this pathophysiologic explanation seems applicable to patients with ACLD, further studies investigating the mechanistic link between bacterial translocation, systemic inflammation and (ROTEM-based assessment of) hemostasis are warranted.

Our study has some limitations: First, we focused on the influence of portal hypertension on ROTEM results in clinically stable outpatients. Therefore, clinical outcome parameters such as bleeding, the subsequent occurrence of (further) hepatic decompensation, thrombo-embolic events, transplantation, or mortality were not assessed. Second, the study might be underpowered for some analyses and may suffer from type 2 error despite including 159 patients. Third, we did not rule out dysfibrinogenemia in our cohort, which could be a reason for delayed clot initiation and attenuated firmness despite sufficiently high fibrinogen levels.

In conclusion, our systemic ROTEM measurements demonstrated prolonged clot formation time and impaired clot firmness in patients with Child–Pugh stage B/C liver dysfunction. Interestingly, portal hypertension impacted ROTEM results only in compensated ACLD patients, with impaired clot firmness when CSPH is present. Mechanistically, bacterial translocation, and systemic inflammation may potentially trigger a procoagulant state in patients with ACLD.

## Electronic supplementary material

Below is the link to the electronic supplementary material.

Supplementary material 1 (DOCX 444 kb)
