# Supplementary material for: Thromboelastometry in patients with advanced chronic liver disease stratified by severity of portal hypertension
Source: Hepatol Int. 2020 Sep 30;14(6):1083–92. doi: 10.1007/s12072-020-10093-3 (PMC7803675; doi:10.1007/s12072-020-10093-3)
Supplement: Supplementary file 1 — Supplementary material 1 (DOCX 444 kb) [file 12072_2020_10093_MOESM1_ESM.docx]

**Supplementary Material**

Thromboelastometry in patients with advanced chronic liver disease stratified by severity of portal hypertension.

Pierre Raeven^1^, Joanna Baron-Stefaniak^1^, Benedikt Simbrunner^2,3^, Alexander Stadlmann^2,3^, Philipp Schwabl^2,3^, Bernhard Scheiner^2,3^, Eva Schaden^1^, Ernst Eigenbauer^4^, Peter Quehenberger^5^, Mattias Mandorfer^2,3^, David Marek Baron^1^, Thomas Reiberger^2,3^

^1^ Department of Anaesthesia, Intensive Care Medicine and Pain Medicine, Medical University of Vienna, Vienna, Austria

^2^ Vienna Hepatic Hemodynamic Lab, Medical University of Vienna, Vienna, Austria

^3^ Division of Gastroenterology and Hepatology, Department of Medicine III, Medical University of Vienna, Vienna, Austria

^4^ IT4Science, Medical University of Vienna, Vienna, Austria

^5^ Department of Laboratory Medicine, Medical University of Vienna, Vienna, Austria

**Table S1.** Contingency table of HVPG strata and Child-Pugh stages.

|  | Child-Pugh-A | Child-Pugh-B | Child-Pugh-C | Total |
| --- | --- | --- | --- | --- |
| HVPG 6-9 mmHg | 15 | 5 | 1 | 21 |
| HVPG 10-19 mmHg | 52 | 26 | 6 | 84 |
| HVPG ≥20 mmHg | 17 | 23 | 14 | 54 |
| Total | 84 | 54 | 21 | 159 |

**Table S2.** Results of selected ROTEM tests in decompensated (Child-Pugh B/C) patients stratified by severity of portal hypertension.^2^

|  | **HVPG** | | |  |
| --- | --- | --- | --- | --- |
|  | **6-9 mmHg** | **10-19 mmHg** | **≥20 mmHg** | **p-value** |
| **EXTEM CT (s)** | 81 [75-84] | 71 [66-83] | 74 [65-85] | 0.373 |
| **EXTEM CFT (s)** | 192 [115-370] | 140 [88-197] | 136 [86-218] | 0.332 |
| **EXTEM MCF (mm)** | 46 [37-58] | 55 [45-61] | 53 [44-61] | 0.337 |
| **INTEM CT (s)** | 226 [200-270] | 205 [169-240] | 203 [181-223] | 0.225 |
| **INTEM CFT (s)** | 151 [97-290] | 112 [78-171] | 112 [70-186] | 0.401 |
| **INTEM MCF (mm)** | 48 [36-55] | 54 [44-60] | 54 [44-60] | 0.386 |
| **FIBTEM MCF (mm)** | 11 [6-14] | 15 [10-21] | 14 [10-19] | 0.228 |
| **Fibrinogen (mg/dl)** | 167 [138-266] | 251 [202-314] | 252 [181-351] | 0.217 |
| **Platelets (G/µl)** | 74 [47-147] | 99 [73-142] | 103 [62-137] | 0.555 |

^2^Data are expressed as medians with interquartile range in square brackets. N=6 (HVPG 6-9 mmHg), n=32 (HVPG 10-19 mmHg, except n=31 for platelet count), n=37 (HVPG≥20 mmHg). Data were analyzed using analysis of variance (ANOVA) with Tukey’s test for post-hoc comparisons (EXTEM MCF, INTEM MCF) or Kruskal-Wallis test with Dunn’s test for post-hoc comparisons (remaining parameters). ROTEM, rotational thromboelastometry; HVPG, hepatic venous pressure gradient; EXTEM, extrinsic thromboelastometry; CT, clotting time; CFT, clot formation time; MCF, maximal clot firmness; INTEM, intrinsic thromboelastometry; FIBTEM, fibrinogen thromboelastometry.

**Table S3.** Contingency table of HVPG strata grades and Child-Pugh class of patients on non-selective betablocker therapy.

|  | Child-Pugh-A | Child-Pugh-B | Child-Pugh-C | Total |
| --- | --- | --- | --- | --- |
| HVPG 6-9 mmHg | 3 | 2 | 0 | 5 |
| HVPG 10-19 mmHg | 12 | 2 | 1 | 15 |
| HVPG ≥20 mmHg | 2 | 5 | 3 | 10 |
| Total | 17 | 9 | 4 | 30 |

**Table S4.** Results of selected ROTEM tests, platelet counts and fibrinogen levels in patients on non-selective betablockers stratified by severity of portal hypertension.^4^

|  | **HVPG** | | |  |
| --- | --- | --- | --- | --- |
|  | **6-9 mmHg** | **10-19 mmHg** | **≥20 mmHg** | **p-value** |
| **EXTEM CT (s)** | 76 [69 – 89] | 73 [66 – 92] | 76 [72 – 82] | 0.921 |
| **EXTEM CFT (s)** | 132 [105 – 287] | 123 [86 – 218] | 157 [87 – 226] | 0.871 |
| **EXTEM MCF (mm)** | 55 [45 – 60] | 55 [48 – 61] | 50 [43 – 60] | 0.579 |
| **INTEM CT (s)** | 227 [199 – 246] | 196 [154 – 228] | 189 [178 – 236] | 0.561 |
| **INTEM CFT (s)** | 118 [91 – 238] | 105 [84 – 182] | 131 [72 – 209] | 0.892 |
| **INTEM MCF (mm)** | 55 [52 – 64] | 55 [45 – 59] | 52 [44 – 59] | 0.474 |
| **FIBTEM MCF (mm)** | 15 [9 – 18] | 13 [10 – 20] | 11 [10 – 16] | 0.727 |
| **Fibrinogen (mg/dl)** | 313 [226 – 370] | 286 [214 – 372] | 235 [172 – 315] | 0.537 |
| **Platelets (G/µl)** | 88 [71 –120] | 100 [58 – 142] | 87 [56 – 117] | 0.734 |

^4^Data are expressed as medians with interquartile range in square brackets. N=5 (HVPG 6-9 mmHg), n=15 (HVPG 10-19 mmHg), n=10 (HVPG≥20 mmHg, except n=9 for platelet count). Data were analyzed using analysis of variance (ANOVA) with Tukey’s test for post-hoc comparisons (EXTEM MCF, INTEM MCF, fibrinogen and platelet count) or Kruskal-Wallis test with Dunn’s for test for post-hoc comparisons (remaining parameters). ROTEM, rotational thromboelastometry; HVPG, hepatic venous pressure gradient; EXTEM, extrinsic thromboelastometry; CT, clotting time; CFT, clot formation time; MCF, maximal clot firmness; INTEM, intrinsic thromboelastometry; FIBTEM, fibrinogen thromboelastometry.

**Table S5.** Results of selected ROTEM tests, platelet counts and fibrinogen levels in Child-Pugh A patients stratified by prognostic stages of ACLD.^5^

|  | **Stage 0 (n=10)** | **Stage 1 (n=19)** | **Stage 2 (n=55)** | **p-value** |
| --- | --- | --- | --- | --- |
| **EXTEM CT (s)** | 85 [68 – 93] | 73 [68 – 77] | 75 [66 – 89] | 0.473 |
| **EXTEM CFT (s)** | 109 [75 – 170] | 121 [98 – 185] | 123 [103 – 217] | 0.297 |
| **EXTEM MCF (mm)** | 59 [52 – 69] | 56 [49 – 60] | 55 [47 – 59] | 0.125 |
| **INTEM CT (s)** | 172 [151 – 197] | 191 [173 – 216] | 198 [170 – 236] * | 0.046 |
| **INTEM CFT (s)** | 102 [60 – 135] | 101 [80 – 135] | 118 [84 – 177] | 0.195 |
| **INTEM MCF (mm)** | 59 [53 – 68] | 55 [50 – 59] | 53 [46 – 57] | 0.056 |
| **FIBTEM MCF (mm)** | 16 [12 – 25] | 12 [11 – 13] | 15 [11 – 18] | 0.191 |
| **Fibrinogen (mg/dl)** | 342 [248 – 446] | 253 [227 – 386] | 295 [238 – 335] | 0.068 |
| **Platelet count (G/µl)** | 155 [124 –179] | 115 [80 –135] * | 84 [58 –120] *** | <0.001 |

^5^Parameters were analyzed based on three prognostic stages in compensated ACLD: stage 0 (HVPG 6-9 mmHg without varices), stage 1 (HVPG≥9 mmHg without varices), and stage 2 (presence of varices). Data are expressed as medians with interquartile range in square brackets. Data were analyzed using analysis of variance (ANOVA) with Tukey’s test for post-hoc comparisons (INTEM CT, platelet count) or Kruskal-Wallis test with Dunn’s test for post-hoc comparisons (remaining parameters). ROTEM, rotational thromboelastometry; ACLD, advanced chronic liver disease; HVPG, hepatic venous pressure gradient; EXTEM, extrinsic thromboelastometry; INTEM, intrinsic thromboelastometry; CT, clotting time; CFT, clot formation time; MCF, maximal clot firmness; FIBTEM, fibrinogen thromboelastometry. *p<0.05 vs. stage 0, ***p<0.001 vs. stage 0.

**Table S6.** ROTEM in patients at high risk of bleeding.^6^

|  | **HVPG** | |  |
| --- | --- | --- | --- |
|  | **6-19 mmHg** | **≥20 mmHg** | **p-value** |
| **EXTEM CT (s)** | 76 [67-83] | 74 [65-85] | 0.990 |
| **EXTEM CFT (s)** | 141 [95-213] | 136 [86-218] | 0.679 |
| **EXTEM MCF (mm)** | 53 [43-60] | 53 [44-61] | 0.985 |
| **INTEM CT (s)** | 207 [170-240] | 203 [181-223] | 0.468 |
| **INTEM CFT (s)** | 116 [81-175] | 112 [70-186] | 0.782 |
| **INTEM MCF (mm)** | 52 [44-59] | 52 [44-61] | 0.840 |
| **FIBTEM MCF (mm)** | 14 [9-19] | 14 [10-19] | 0.947 |
| **Fibrinogen (mg/dl)** | 239 [186-310] | 252 [181-351] | 0.656 |
| **Platelets (G/µl)** | 96 [67-138] | 103 [62-137] | 0.677 |

^6^ROTEM values from patients with Child-Pugh B and C (merged) who were stratified based on the presence or absence of high-risk portal hypertension, as defined by HVPG≥20 mmHg. Data are expressed as medians with interquartile range in square brackets. N=38 (HVPG 6-19 mmHg, except n=37 for platelet count), n=37 (HVPG≥20 mmHg). Data were analyzed using the unpaired Student’s t-test (INTEM MCF) or Mann-Whitney U test (remaining parameters). ROTEM, rotational thromboelastometry; HVPG, hepatic venous pressure gradient; EXTEM, extrinsic thromboelastometry; CT, clotting time; CFT, clot formation time; MCF, maximal clot firmness; INTEM, intrinsic thromboelastometry; FIBTEM, fibrinogen thromboelastometry.

**Table S7.** Correlation of clinical scores and laboratory parameters to ROTEM parameters in the overall cohort.^7^

|  | **EXTEM** | | | **INTEM** | | | **FIBTEM** |
| --- | --- | --- | --- | --- | --- | --- | --- |
|  | **CT** | **CFT** | **MCF** | **CT** | **CFT** | **MCF** | **MCF** |
| **HVPG** | -0.022 | 0.008 | -0.107 | 0.031 | 0.022 | -0.125 | -0.015 |
| **Liver stiffness** | 0.635 | 0.034^*^ | 0.365 | 0.380 | 0.021^*^ | 0.530 | 0.113 |
| **Child-Pugh score** | 0.066 | 0.098 | -0.178^*^ | 0.175^*^ | 0.069 | -0.188^*^ | -0.188^*^ |
| **MELD-Na** | 0.059 | 0.026 | -0.126 | 0.155 | 0.009 | -0.149 | -0.068 |
| **MELD-UNOS** | 0.091 | 0.084 | -0.183^*^ | 0.218^*^ | 0.076 | -0.215^*^ | -0.150 |
| **Sodium (mmol/l)** | 0.100 | 0.271^*^ | -0.245^*^ | 0.135^*^ | 0.284^*^ | -0.216^*^ | -0.252^*^ |
| **Creatinine (mg/dl)** | -0.224^*^ | -0.172^*^ | 0.174^*^ | -0.247^*^ | -0.194^*^ | 0.179^*^ | 0.197^*^ |
| **Bilirubin (mg/dl)** | 0.151 | 0.216^*^ | -0.310^*^ | 0.323^*^ | 0.204^*^ | -0.336^*^ | -0.284^*^ |
| **Albumin (g/l)** | 0.009 | -0.078 | 0.155 | -0.048 | -0.026 | 0.151^*^ | 0.115 |
| **PT-INR** | 0.184^*^ | 0.340^*^ | -0.415^*^ | 0.386^*^ | 0.322^*^ | -0.428^*^ | -0.409^*^ |
| **aPTT (s)** | 0.070 | 0.153 | -0.252^*^ | 0.460^*^ | 0.132 | -0.282^*^ | -0.202^*^ |
| **Fibrinogen (mg/dl)** | -0.168^*^ | -0.745^***^ | 0.795^***^ | -0.204^*^ | -0.712^***^ | 0.759^***^ | 0.899^***^ |
| **Platelet count (G/l)** | -0.102 | -0.780^***^ | 0.831^***^ | -0.275^***^ | -0.795^***^ | 0.837^***^ | 0.555^***^ |
| **vWF antigen (%)** | 0.859 | 0.722 | 0.628 | 0.521 | 0.507 | 0.382 | 0.656 |
| **ASAT (U/l)** | 0.091 | 0.060 | -0.094 | 0.123 | 0.036 | -0.113 | -0.089 |
| **ALAT (U/l)** | 0.124 | 0.053 | -0.091 | -0.005 | 0.048 | -0.105 | -0.121 |
| **ASAT/ALAT** | 0.002 | -0.033 | 0.025 | 0.162 | -0.046 | 0.030 | 0.073 |
| **Gamma-GT (U/l)** | 0.026 | -0.315 | 0.275 | -0.140 | -0.304 | 0.267 | 0.297 |
| **Hemoglobin (g/dl)** | 0.195^*^ | 0.061 | -0.088 | 0.061 | 0.093 | -0.091 | -0.173^*^ |
| **Hematocrit (%)** | 0.206^*^ | 0.025 | -0.046 | 0.038 | 0.063 | -0.050 | -0.134 |
| **LBP (µg/ml)** | 0.171^*^ | -0.426^***^ | 0.494^***^ | -0.199^*^ | -0.409^***^ | 0.498^***^ | 0.541^***^ |
| **Leukocyte count (G/l)** | -0.003 | -0.610^***^ | 0.595^***^ | -0.116 | -0.599^***^ | 0.576^***^ | 0.412^***^ |
| **CRP (mg/dl)** | 0.168^*^ | -0.305^***^ | 0.256^**^ | -0.076 | -0.314^***^ | 0.247^**^ | 0.305^***^ |
| **Interleukin-6 (pg/ml)** | 0.116 | -0.062 | 0.067 | -0.001 | -0.113 | 0.087 | 0.048 |
| **Procalcitonin (ng/ml)** | -0.049 | -0.195^*^ | 0.134 | 0.152 | -0.216^**^ | 0.117 | 0.194^*^ |

^7^Numbers indicate Spearman's rank correlation coefficient (rho). *P<0.05, **P<0.01, ***P<0.001. Abbreviations: CT, clotting time; CFT, clot formation time; MCF, maximum clot firmness, HVPG, hepatic venous pressure gradient, MELD, Model for End-stage Liver Disease; UNOS, United Network for Organ Sharing; PT-INR, prothrombin international normalized ratio; aPTT, activated partial thromboplastin time; vWF, von Willebrand factor; ASAT, aspartate aminotransferase; ALAT, alanine transaminase; gamma-GT, gamma-glutamyltransferase; LBP, lipopolysaccharide-binding protein; CRP, C-reactive protein.

**Table S8.** Parameters of bacterial translocation and systemic inflammation in all patients at high risk of bleeding.^8^

|  | **HVPG** | |  |
| --- | --- | --- | --- |
|  | **6-19 mmHg** | **≥20 mmHg** | **p-value** |
| **LBP (mcg/ml)** | 7.00 [5.18-8.51] (n=103) | 6.71 [4.98-9.95] (n=54) | 0.911 |
| **Leukocytes (G/l)** | 4.17 [3.11-5.95] (n=104) | 5.21 [3.31-6.25] (n=54) | 0.430 |
| **CRP (mg/dl)** | 0.26 [0.11-0.57] (n=105) | 0.54 [0.21-1.20] (n=54) | 0.003 |
| **Procalcitonin (ng/ml)** | 0.08 [0.04-0.16] (n=102) | 0.12 [0.07-0.23] (n=53) | 0.451 |

^8^ Parameters of bacterial translocation and systemic inflammation ROTEM values from all patients who were stratified based on the presence or absence of high-risk portal hypertension, as defined by HVPG≥20 mmHg. Data are expressed as medians with interquartile range in square brackets. Data were analyzed using the Mann-Whitney U test. HVPG, hepatic venous pressure gradient; LBP, lipopolysaccharide-binding protein; CRP, C-reactive protein.

**Table S9.** Parameters of bacterial translocation and systemic inflammation in Child-Pugh B and C patients at high risk of bleeding.^9^

|  | **HVPG** | |  |
| --- | --- | --- | --- |
|  | **6-19 mmHg** | **≥20 mmHg** | **p-value** |
| **LBP (mcg/ml)** | 6.53 [4.70-8.64] (n=38) | 7.01 [4.94-11.60] (n=37) | 0.143 |
| **Leukocytes (G/l)** | 4.11 [3.16-5.22] (n=37) | 5.37 [3.48-6.43] (n=37) | 0.432 |
| **CRP (mg/dl)** | 0.49 [0.19-1.30] (n=38) | 0.77 [0.24-2.23] (n=37) | 0.504 |
| **Procalcitonin (ng/ml)** | 0.11 [0.68-0.18] (n=38) | 0.15 [0.08-0.26] (n=36) | 0.141 |

^9^ Parameters of bacterial translocation and systemic inflammation ROTEM values from all patients who were stratified based on the presence or absence of high-risk portal hypertension, as defined by HVPG≥20 mmHg. Data are expressed as medians with interquartile range in square brackets. Data were analyzed using the unpaired t test (LBP) or the Mann-Whitney U test (remaining parameters). HVPG, hepatic venous pressure gradient; LBP, lipopolysaccharide-binding protein; CRP, C-reactive protein.

**Fig. S1.** Association of Impact of bacterial translocation and systemic inflammation on intrinsic and fibrinogen thromboelastometry (INTEM/FIBTEM) results.^10^

****^10^LBP (A), leukocytes (B), CRP (C) and procalcitonin (D) in INTEM CT strata. LBP (E), leukocytes (F), CRP (G) and procalcitonin (H) in INTEM CFT strata. LBP (I), leukocytes (J), CRP (K) and procalcitonin (L) in INTEM MCF strata. LBP (M), leukocytes (N), CRP (O) and procalcitonin (P) in FIBTEM MCF strata. *p<0.05; **p<0.01; ***p<0.001 (ANOVA/Tukey’s or Kruskal-Wallis/Dunn’s). Dotted lines indicate (lower and) upper refence value according to manufacturer/laboratory. INTEM, intrinsic thromboelastometry; CT, clotting time; CFT, clot formation time; MCF, maximal clot firmness; FIBTEM, fibrinogen thromboelastometry. LPB, lipopolysaccharide-binding protein; CRP, C-reactive protein; Q1, 0-20 percentile; Q2-4, 21-80 percentile; Q5, 81-100 percentile.
